# Melatonin Sensitizes Hepatocellular Carcinoma Cells to Chemotherapy Through Long Non-Coding RNA RAD51-AS1-Mediated Suppression of DNA Repair

**DOI:** 10.3390/cancers10090320

**Published:** 2018-09-10

**Authors:** Chin-Chuan Chen, Chi-Yuan Chen, Shu-Huei Wang, Chau-Ting Yeh, Shih-Chi Su, Shir-Hwa Ueng, Wen-Yu Chuang, Chuen Hsueh, Tong-Hong Wang

**Affiliations:** 1 Tissue Bank, Chang Gung Memorial Hospital, Tao-Yuan 33305, Taiwan; chinchuan@mail.cgu.edu.tw (C.-C.C.); d49417002@gmail.com (C.-Y.C.); ch9211@cgmh.org.tw (C.H.); 2 Graduate Institute of Natural Products, Chang Gung University, Tao-Yuan 33303, Taiwan; 3 Graduate Institute of Health Industry Technology and Research Center for Industry of Human Ecology, College of Human Ecology, Chang Gung University of Science and Technology, Tao-Yuan 33303, Taiwan; 4 Department of Anatomy and Cell Biology, College of Medicine, National Taiwan University, Taipei 10617, Taiwan; shwang@ntu.edu.tw; 5 Liver Research Center, Department of Hepato-Gastroenterology, Chang Gung Memorial Hospital, Tao-Yuan 33305, Taiwan; chauting@adm.cgmh.org.tw; 6 Whole-Genome Research Core Laboratory of Human Diseases, Chang Gung Memorial Hospital, Keelung 20401, Taiwan; zenith5862@hotmail.com; 7 Department of Anatomic Pathology, Chang Gung Memorial Hospital, Chang Gung University School of Medicine, Tao-Yuan 33305, Taiwan; shu922@adm.cgmh.org.tw (S.-H.U.); s12126@cgmh.org.tw (W.-Y.C.)

**Keywords:** melatonin, hepatocellular carcinoma, DNA repair, lncRNA-RAD51-AS1, RAD51

## Abstract

DNA repair systems are abnormally active in most hepatocellular carcinoma (HCC) cells due to accumulated mutations, resulting in elevated DNA repair capacity and resistance to chemotherapy and radiotherapy. Thus, targeting DNA repair mechanisms is a common treatment approach in HCC to sensitize cancer cells to DNA damage. In this study, we examined the anti-HCC effects of melatonin and elucidated the regulatory mechanisms. The results of functional assays showed that in addition to inhibiting the proliferation, migration, and invasion abilities of HCC cells, melatonin suppressed their DNA repair capacity, thereby promoting the cytotoxicity of chemotherapy and radiotherapy. Whole-transcriptome and gain- and loss-of-function analyses revealed that melatonin induces expression of the long noncoding RNA RAD51-AS1, which binds to RAD51 mRNA to inhibit its translation, effectively decreasing the DNA repair capacity of HCC cells and increasing their sensitivity to chemotherapy and radiotherapy. Animal models further demonstrated that a combination of melatonin and the chemotherapeutic agent etoposide (VP16) can significantly enhance tumor growth inhibition compared with monotherapy. Our results show that melatonin is a potential adjuvant treatment for chemotherapy and radiotherapy in HCC.

## 1. Introduction

Hepatocellular carcinoma (HCC) is the most common cancer of the liver and ranks fifth in global cancer incidence [1,2], with ~700,000 people worldwide being diagnosed with HCC every year. Moreover, HCC is one of the most refractory malignant tumors, especially when it has progressed to late stages and surgical resection is no longer feasible, and chemotherapy is the only treatment option available for these patients. Etoposide (VP16) and camptothecin (CPT), which induce DNA damage in cancer cells and result in apoptosis, are commonly used in the clinic as chemotherapeutic agents [3,4]. However, DNA repair systems in most HCC cells are abnormally active due to the accumulation of mutations, resulting in elevated DNA repair capacity and poor chemotherapy outcomes. Recent studies have demonstrated that the effects of chemotherapy and radiotherapy can be significantly enhanced when the DNA repair pathway in cancer cells is blocked, and accordingly, DNA repair blockers, such as trans-resveratrol, B02, and IBR2, have been widely used for clinical treatment to reduce the dose of chemotherapeutic drugs and improve their therapeutic effects [5,6,7].

DNA damage repair is an important mechanism for maintaining chromosome stability [8,9], and dysregulation of proteins involved in DNA repair increases the probability of DNA mutations and can result in cell death or oncogenesis [10,11]. DNA repair mechanisms can be subdivided into two major categories: double-strand break repair (DSBR) and single-strand break repair (SSBR) [12]. Homologous recombination (HR) is the major mechanism of DSBR, and RAD51 is the key protein involved in this process [13]. The RAD51 protein binds to single-stranded DNA to promote HR to complete DNA repair, and suppressing RAD51 expression strongly inhibits the DNA repair process [14]. Thus, many RAD51 inhibitors, such as (*E*)-3-benzyl-2-(2-(pyridin-3-yl)vinyl)quinazolin-4(3H)-one (i.e., B02) and arsenic trioxide (ATO), have been validated as adjuvant therapies for HCC treatment and have been proven to enhance the effects of chemotherapy and radiotherapy [15,16,17].

Melatonin is the main hormone secreted by the pineal gland in the human brain. In addition to regulating the sleep–wake cycle, melatonin and its metabolites are strong free-radical scavengers that decrease cellular damage arising from the peroxides produced during physiological metabolic processes [18,19]. Moreover, melatonin can activate immunity, inhibit angiogenesis and cell growth, and suppress the metastasis of several cancers [20,21]. Although recent studies have reported that melatonin can inhibit the growth and metastasis of liver cancer cell lines [22], the findings were limited to cellular experiments, and the downstream regulatory mechanisms remain unclear. In addition, further investigation is needed to clarify whether melatonin can serve as an adjuvant treatment for HCC.

Long non-coding RNAs (lncRNAs) are involved in regulating gene expression and protein activity [23,24] and also participate in multiple physiological regulatory and drug effector mechanisms [25,26,27]. Thus far, ~15,000 lncRNA genes have been discovered, yet functions are known for only 1%. In addition, lncRNAs are abnormally expressed in many diseases, indicating their regulatory relationship with pathophysiology [28,29]. As many lncRNAs participate in the effector mechanisms of drugs, they are likely to be involved in melatonin-based effector mechanisms against HCC. In this study, we examined the feasibility of using melatonin for HCC treatment by elucidating the regulatory roles of lncRNA in this process. The results revealed that melatonin can significantly inhibit the proliferation, migration, and invasion capacities of HCC cells and can synergize with chemotherapeutic agents to enhance their cytotoxic effects against HCC cells. This process is mainly mediated through induction of lncRNA RAD51-AS1 expression; the lncRNA binds to RAD51 mRNA to reduce RAD51 protein expression, thereby suppressing the DNA damage repair capacity of HCC cells. Our in vivo animal experiments also demonstrated significantly enhanced cytotoxic effects of etoposide on HCC cell lines when melatonin was administered in combination with the chemotherapeutic agent etoposide.

## 2. Results

### 2.1. Melatonin Inhibits the Proliferation, Migration, and Invasion Abilities of HCC Cells

**Figure 1:**
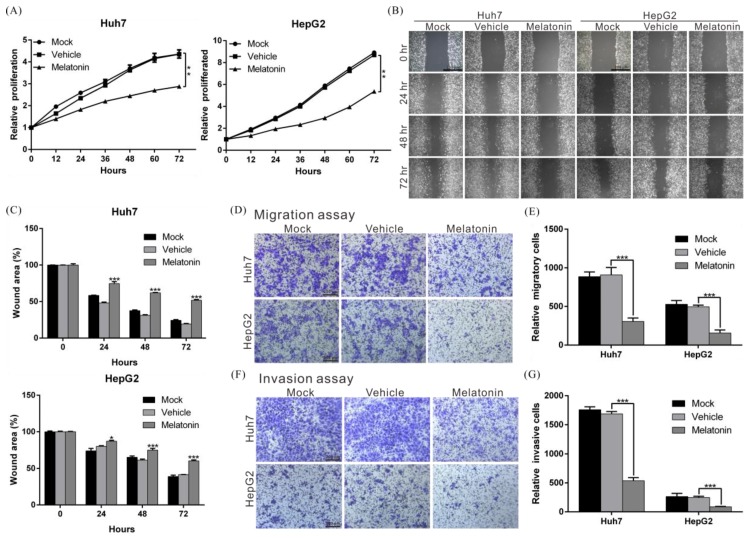
Melatonin suppressed proliferation, migration and invasion capacities in HCC cells. (**A**) Huh7 and HepG2 cells were treated with 1 mM melatonin, and cell proliferation capacity was assessed at the indicated time points using an xCELLigence real-time cell analyzer. Mock: cells treated with DMEM medium. Vehicle: cells treated with DMSO. *p* < 0.01 (**), as assessed using Student’s *t*-test. (**B**) Wound-healing abilities of Huh7 and HepG2 cells treated with/without 1 mM melatonin were compared. Quantification of the cell wound-healing assays is presented in (**C**). *p* < 0.05 (*), *p* < 0.001 (***). (**D**) The migration capacities of Huh7 and HepG2 cells treated with/without 1 mM melatonin were compared using a transwell assay. Quantitative cell migration assay results are shown in (**E**). Data represent the mean ± S.D. of three independent experiments. *p* < 0.001 (***). (**F**) Invasion capacities of Huh7 and HepG2 cells were measured using Matrigel-coated polyethylene terephthalate membrane inserts. Quantification of the cell invasion assay is shown in (**G**). *p* < 0.001 (***). All experiments were performed in triplicate.

To understand whether melatonin has therapeutic effects on HCC, HCC cell lines HepG2 and Huh7 (which exhibit higher expression of the melatonin receptor; Supplementary Figure S1A,B) were treated with different concentrations of melatonin. The inhibitory effects of melatonin on cell growth began at 0.1 mM, and these effects were dose dependent (Supplementary Figure S1C). The half-maximal inhibitory concentration (IC_50_) of melatonin was approximately 1 mM, i.e., cell growth was significantly inhibited when HepG2 and Huh7 HCC cells were exposed to 1 mM melatonin. Compared with the control group treated with vehicle (DMSO), HepG2 and Huh7 cells treated with melatonin for 72 h showed growth rate inhibition of 38 and 33%, respectively (Figure 1A). Similar results were obtained in colony formation assays (Supplementary Figure S2), whereby melatonin significantly inhibited the ability of HepG2 and Huh7 cells to form colonies. The above results indicate that melatonin inhibits HCC cell proliferation.

The metastatic and invasive properties of cancer cells contribute to treatment resistance. To elucidate whether melatonin affects these properties of HCC cells, we treated cells with 1 mM melatonin and performed transwell and wound-healing assays to analyze cell migration status. According to the results, melatonin maximally inhibited cell migration capacity by 66% (Figure 1B–E). In the invasion assay, melatonin suppressed the invasiveness of HepG2 and Huh7 cells by 64 and 68%, respectively (Figure 1F,G). The above findings show that melatonin exerts inhibitory effects on HCC cells.

### 2.2. Melatonin Increases the Sensitivity of HCC Cells to Chemotherapy and Radiotherapy

**Figure 2:**
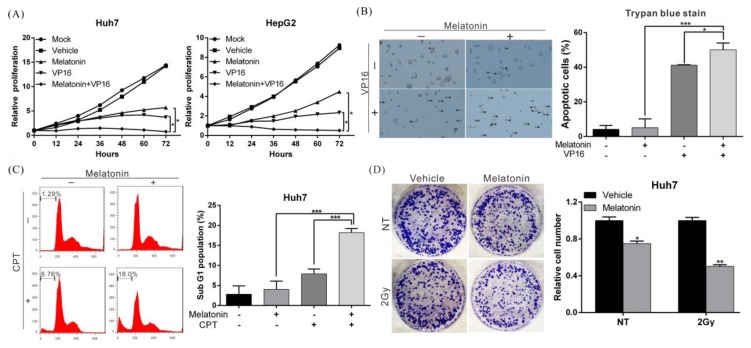
Melatonin enhanced the sensitivity of HCC cells to chemotherapy and radiotherapy. (**A**) The proliferation capacity of Huh7 and HepG2 cells treated with 1 mM melatonin, 200 µM etoposide (VP16), or both was monitored using an xCELLigence real-time cell analyzer. *p* < 0.05 (*), as assessed using Student’s *t*-test. (**B**,**C**) An trypan blue exclusion assay and flow cytometry showed that combined treatment with 1 mM melatonin significantly increased the cytotoxicity of 200 µM etoposide (VP16) and 1 µM camptothecin (CPT) in Huh7 cells. Data are expressed as the mean ± S.D. of three independent experiments. The arrows indicate the apoptotic cells. (**D**) Effect of melatonin on the radiosensitivity of Huh7 cells. Cells were irradiated with Cs-137 at a dose of 2 Gy, followed by treated with/without 1 mM melatonin for 24 h and then cultured for an additional 10 days in the absence of melatonin (left panel). The numbers of foci were counted, and the results are presented in the right panel. *p* < 0.05 (*), *p* < 0.01 (**), *p* < 0.001 (***). All experiments were performed in triplicate.

To further clarify the therapeutic effects of melatonin in combination with other anticancer treatments [4], we treated HCC cells with melatonin and the chemotherapeutic agent etoposide (VP16) and compared effects on growth inhibition with those after single-drug treatment. Compared with etoposide alone, combined treatment with melatonin significantly enhanced inhibitory effects on HepG2 and Huh7 cell growth (Figure 2A). In a trypan blue exclusion assay, combined treatment with melatonin significantly enhanced the cytotoxicity of etoposide in HCC cells compared to the drug alone, with the proportion of apoptotic Huh7 cells increasing by 22% (Figure 2B). Similar results were obtained in MTT (3-[4,5-dimethylthiazol-2-yl]-2,5 diphenyltetrazolium bromide) assay and TUNEL assay (Supplementary Figure S3). Figure 2C shows that similar results were obtained by flow cytometry when etoposide was replaced with the chemotherapeutic drug camptothecin (CPT). Additionally, suppression of colony formation increased by 25% when HCC cell lines were exposed to both melatonin and irradiation compared with radiation alone (Figure 2D). These data show that melatonin can increase the sensitivity of HCC cells to chemotherapeutic drugs as well as radiotherapy.

### 2.3. Melatonin Inhibits the Growth of HCC Tumors and Increases the In Vivo Inhibitory Effects of Chemotherapeutic Drugs on Tumors

**Figure 3:**
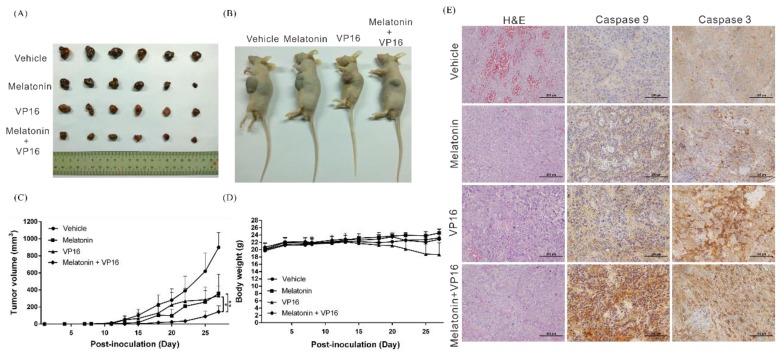
Melatonin suppressed tumor growth and enhanced etoposide (VP16)-induced inhibitory effects on tumors in vivo. (**A**,**B**) A total of 5 × 10^6^ Huh7 cells were injected subcutaneously into both the right and left flanks of nude mice (*n* = 6, each group). Representative images show the tumor xenografts after 4 weeks. (**C**) Tumor volumes were measured three times a week and calculated using the formula: length × width^2^ × 0.5. Bars indicate S.D. * *p* < 0.05, ** *p* < 0.01. (**D**) Body weights were recorded three times a week. (**E**) Histological analysis of xenografts using H&E staining and immunohistochemical staining for cleaved caspase-3 and cleaved caspase-9 showed that melatonin enhanced etoposide-induced apoptosis. Magnification: 400×, scale bar = 100 μm.

To verify the experimental results described above, we used a mouse xenograft model to evaluate the inhibitory effects of melatonin on tumor growth in vivo. The results indicated that compared with the control group receiving only vehicle (DMSO), treatment with melatonin or etoposide alone significantly inhibited the growth of tumors. When melatonin was used in combination with etoposide, the inhibitory effect on tumor growth was more than 50% greater than that of each drug alone (Figure 3A–C), which was consistent with the results of the in vitro cellular experiments. In addition, melatonin injection did not significantly affect the weight of the mice during the experimental period (Figure 3D), suggesting that melatonin is not toxic to mice. We then performed hematoxylin and eosin (H&E) staining and immunohistochemical analysis of tumor tissues and found that compared with etoposide treatment alone, tumor tissues simultaneously treated with melatonin and etoposide showed significantly higher caspase-9 and caspase-3 expression (Figure 3E). This finding suggests that melatonin enhances etoposide-induced apoptosis.

### 2.4. Melatonin Decreases the DNA Damage Repair Capacity of HCC Cells by Inhibiting RAD51 Expression

**Figure 4:**
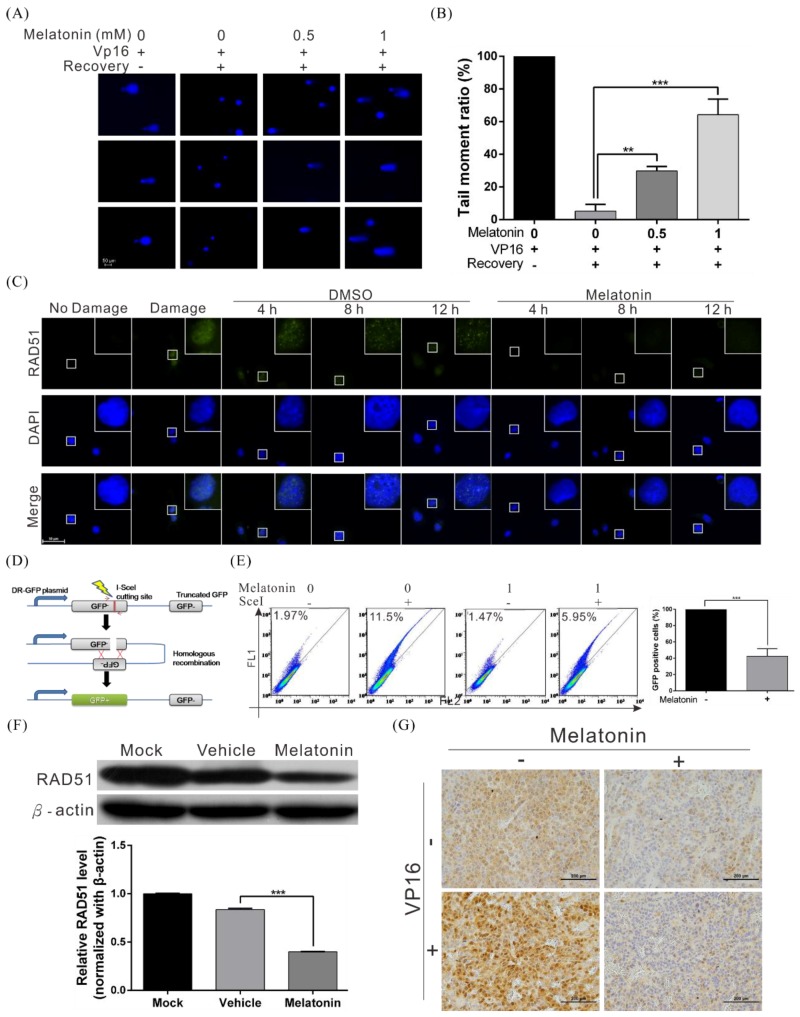
Melatonin suppressed the DNA repair capacity of HCC cells by inhibiting RAD51 expression. (**A**) A comet assay shows DNA repair activity after treatment with melatonin. Huh7 cells were treated with 200 µM etoposide (VP16) for 1 h, followed by treatment with different concentrations of melatonin in etoposide-free medium for 4 h. The cells were harvested and subjected to comet assays to detect DNA repair activity. The rows of panels present the results of three individual experiments. Quantification of cell repair activity is shown in (**B**). *p* < 0.01 (**), *p* < 0.001 (***). (**C**) Huh7 cells were treated with 200 μM etoposide (VP16) for 1 h to induce DNA damage, followed by recovery in medium with or without 1 mM melatonin. Cells were processed for immunofluorescence staining at various time points to detect the formation of Rad51 foci (green). Nuclei were stained with DAPI (blue). (**D**) A schematic representing the principle of the HR reporter assay. The DR-GFP system, which contains two mutated GFP genes (termed GFP^−^) was applied to detect HR repair in human cancer cells. As the left GFP^-^ gene contains an I-*Sce*I endonuclease site, expression of I-*Sce*I leads to a DSB that can be repaired by HR using the homologous region in the truncated GFP gene. The complete HR results in expression of a functional GFP (GFP^+^) that can be detected by flow cytometry. (**E**) The results of flow cytometry indicate that melatonin significantly inhibited HR in HCC cells. (**F**) Western blot analysis of RAD51 in Huh7 cells treated with or without 1 mM melatonin for 48 h; β-actin was included as an internal control (upper panel). Quantitative results are shown in the lower panel. (**G**) Downregulation of RAD51 in mice xenografts treated with melatonin, as examined by immunohistochemistry.

In both cellular and animal experiments, we observed that melatonin can enhance the cytotoxicity of radiotherapy and chemotherapeutic drugs in HCC cells, in addition to inhibiting the growth, migration, and invasion abilities of HCC cell lines. The main effector mechanisms of radiotherapy and chemotherapy are mediated by inducing cellular DNA damage, which results in apoptosis. To understand whether melatonin is involved in inhibiting DNA damage repair capacity (thereby increasing the sensitivity of cells to chemotherapy and radiotherapy), we used the comet assay to assess whether melatonin affects DNA damage repair in HCC cell lines. We found that the speed of DNA damage repair was significantly slower in cells treated with melatonin than in control group cells (Figure 4A,B). Furthermore, formation of DNA damage-induced RAD51 foci was significantly reduced at every time point after melatonin treatment (Figure 4C), indicating that melatonin can suppress the DNA damage repair capacity of cells.

HR is the most accurate mechanism of DSBR in the cell; thus, we performed an HR reporter assay to determine whether melatonin regulates HR (Figure 4D). The results revealed that melatonin significantly inhibited HR compared with the vehicle control (Figure 4E). 

To understand the mechanism by which melatonin inhibits HR, we used western blotting to analyze expression of HR-associated proteins (Supplementary Figure S4, Figure 4F) and found significant decreases in RAD51, a key protein in HR, after cells were treated with melatonin (Figure 4F). In addition, immunohistochemical staining of mouse tumor tissue slices revealed that melatonin significantly inhibited expression and nuclear transport of RAD51 in tissues (Figure 4G). The above results prove that melatonin can suppress HR in HCC cells by inhibiting RAD51 expression, thereby increasing the cytotoxicity of chemotherapy and radiotherapy.

### 2.5. Melatonin Induces Expression of lncRNA RAD51-AS1, Causing Reduced RAD51 Levels

**Figure 5:**
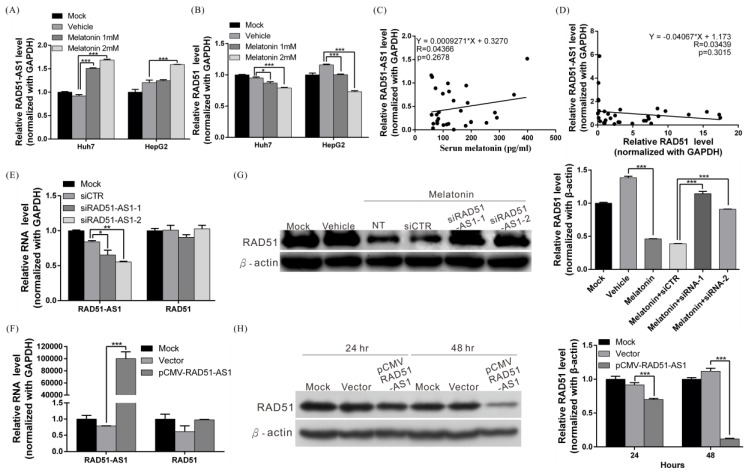
Melatonin induced expression of lncRNA RAD51-AS1, causing RAD51 downregulation. (**A**,**B**) Huh7 and HepG2 cells were treated with 1 mM melatonin for 48 h, and expression of RAD51-AS1 and RAD51 was examined by quantitative real-time RT-PCR. *p* < 0.001 (***) (**C**) Human serum melatonin levels and hepatic RAD51-AS1 levels were measured by ELISA and real-time RT-PCR, respectively (*n* = 33). RAD51-AS1 expression was positively correlated with serum melatonin levels. (**D**) RAD51-AS1 and RAD51 expression in human liver tissues was analyzed by real-time RT-PCR (*n* = 33). RAD51-AS1 expression was negatively correlated with RAD51 expression. (**E**) Real-time RT-PCR results showed that silencing RAD51-AS1 expression had no effect on RAD51 mRNA levels. (**F**) Real-time RT-PCR results showed that overexpression of RAD51-AS1 had no effect on RAD51 mRNA levels. (**G**) Huh7 cells were transfected with 50 nM synthesized random control siRNA (si-CTR) or RAD51-AS1-specific siRNA (siRAD51-AS1) and then cotreated with 1 mM melatonin. After 48 h of treatment, cells were harvested and subjected to western blotting to detect RAD51 levels; β-actin served as an internal control (left panel). Quantitative results are shown in the right panel. *p* < 0.001 (***). (**H**) Huh7 cells were transfected with a RAD51-AS1-expressing plasmid or the empty vector; after 24 h and 48 h, the cells were harvested and subjected to western blotting to examine RAD51 protein levels (left panel). Quantification of the western blotting results are shown in the right panel. *p* < 0.05 (*), *p* < 0.01 (**), *p* < 0.001 (***). All experiments were performed in triplicate.

Recent studies have demonstrated that lncRNAs can act via different mechanisms to regulate gene expression. To examine whether lncRNAs participate in melatonin-mediated RAD51 regulation, whole-transcriptome sequencing was performed to assess lncRNA expression status in Huh7 and HepG2 cells treated with melatonin (Supplementary Figure S5). Expression of a recently discovered lncRNA, RAD51-AS1, was significantly increased after melatonin treatment. We then confirmed this finding by real-time PCR and found that lncRNA RAD51-AS1 expression in cells treated with melatonin increased 1.8-fold compared with that of the control group (vehicle only) and that RAD51 mRNA levels significantly decreased (Figure 5A,B). 

When analyzing clinical samples, we did not find a significant correlation between serum melatonin levels and RAD51-AS1 or RAD51 expression. However, we did observe a positive correlation between serum the melatonin concentration and HCC tissue RAD51-AS1 expression; conversely, RAD51-AS1 levels and RAD51 expression were negatively correlated in HCC tissues (Figure 5C,D). These results suggest the existence of a regulatory relationship among melatonin, lncRNA RAD51-AS1, and RAD51.

Previous studies have confirmed that many antisense lncRNAs can form a double-stranded RNA structure with the sense mRNA strand and that this mechanism can (1) stabilize the mRNA structure and protein expression (2) activate RNA interference mechanisms that degrade the double-stranded RNA and inhibit protein expression or (3) inhibit translation initiation. To determine whether binding of lncRNA RAD51-AS1 to RAD51 mRNA and the corresponding formation of double-stranded RNA can induce mRNA interference and result in RAD51 mRNA degradation, we used siRNA to silence expression of lncRNA RAD51-AS1 and then performed real-time PCR and western blotting to assess RAD51 mRNA and protein expression. After RAD51-AS1 was silenced, RAD51 protein expression increased significantly (Figure 5G). Conversely, overexpression of lncRNA RAD51-AS1 decreased cellular RAD51 protein expression levels by 85% at 48 h (Figure 5H). However, RAD51 mRNA levels were not changed (Figure 5E,F). The above results indicate that lncRNA RAD51-AS1 can suppress RAD51 protein expression, possibly by inhibiting translation initiation.

### 2.6. Melatonin Increases the Sensitivity of HCC Cells to Chemotherapeutic Agents by Inducing Expression of lncRNA RAD51-AS1

**Figure 6:**
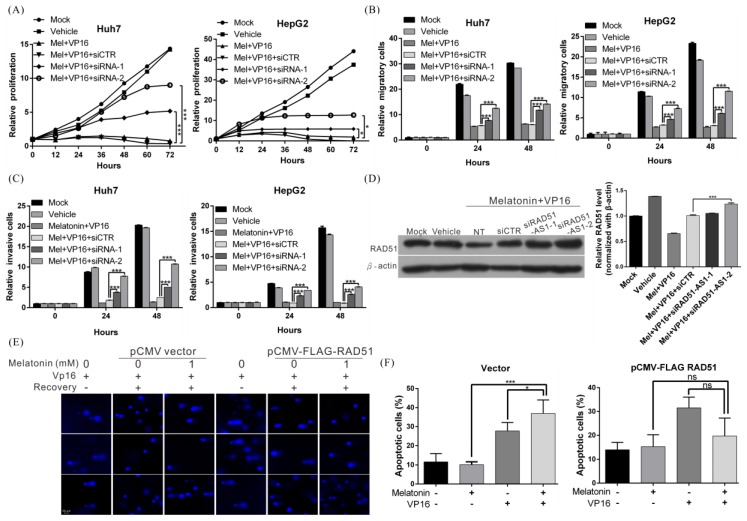
Melatonin enhanced the sensitivity of HCC cells to chemotherapy by inducing lncRNA RAD51-AS1 expression. (**A**–**C**) The effects of melatonin combined with etoposide (VP16) on Huh7 and HepG2 cell proliferation, migration and invasion with/without treatment with RAD51-AS1 siRNA (50 nM). *p* < 0.05 (*), *p* < 0.001 (***) (**D**) Western blot analysis shows the effect on expression of RAD51 in Huh7 cells after the aforementioned treatments (left panel). Quantitative results are shown in the right panel. *p* < 0.001 (***). (**E**) A comet assay showing that overexpression of RAD51 attenuated the melatonin-mediated inhibition of DNA repair. (**F**) An trypan blue exclusion assay showing that melatonin enhanced etoposide-induced apoptosis, whereas the apoptosis ratio was significantly decreased when RAD51 was overexpressed. All experiments were performed in triplicate.

We then conducted a rescue assay to verify whether the anticancer effects of melatonin are mediated by lncRNA RAD51-AS1. Treatment with both melatonin and etoposide significantly inhibited the proliferation, migration, and invasion abilities of cells, and these effects were reversed when expression of lncRNA RAD51-AS1 was silenced during treatment (Figure 6A–C). Base on western blotting, the original RAD51 protein expression, which had been inhibited, was immediately restored after lncRNA RAD-51-AS1 was silenced (Figure 6D). Similarly, when cells overexpressing RAD51 were treated with both melatonin and etoposide, the inhibition of DNA repair by melatonin was attenuated, and the etoposide-induced cellular apoptosis ratio significantly decreased (Figure 6E,F). These findings indicate that the anti-HCC mechanisms of action involving melatonin are due to regulation of RAD51 by lncRNA RAD51-AS1. Specifically, melatonin induces RAD51-AS1 expression, which downregulates RAD51 protein expression. This effect decreased the DNA damage repair capacity of HCC cells, thereby increasing the cytotoxicity of chemotherapeutic agents.

## 3. Discussion

**Figure 7:**
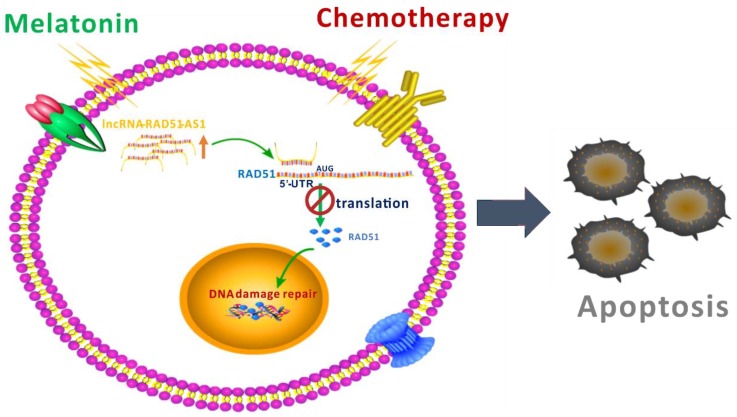
A schematic representation summarizing the mechanism by which melatonin suppresses DNA repair.

DNA repair mechanisms are activated when cells undergo DNA damage, and apoptosis occurs if the DNA cannot be repaired. Nevertheless, most tumor cells accumulate mutations in their DNA repair proteins, resulting in hyperactive DNA repair [30,31,32,33]; even if these cells are damaged by chemotherapeutic drugs, apoptosis will not be invoked [34,35]. This phenomenon is one of the reasons for the resistance of cancer cells to chemotherapy. Therefore, understanding the mechanisms by which cancer cells activate DNA repair and blocking this process are an effective strategy for increasing the therapeutic effectiveness of chemotherapy [36,37,38,39]. In this study, we used cellular and animal model experiments to demonstrate that melatonin can increase the sensitivity of HCC cells to chemotherapy and radiotherapy. Melatonin reduced RAD51 protein expression through lncRNA RAD51-AS1 and decreased DNA damage repair capacity, resulting in an inability to repair DNA damage caused by chemotherapy and radiotherapy and thereby enhancing cell death (Figure 7). To the best of our knowledge, our study is the first to show that melatonin can regulate the lncRNA RAD51-AS1 and thus control a DNA damage repair mechanism.

There have been few studies to date on lncRNA RAD51-AS1, and the exact mechanism of action on cellular physiology has not been reported [40]. One recent study on ovarian cancer revealed that overexpression of RAD51-AS1 can promote cell cycle progression and inhibit apoptosis [40], though the regulatory mechanisms involved were not addressed. Our recent study showed that lncRNA RAD51-AS1 can regulate RAD51 protein expression and thus control DNA damage repair [41]. lncRNA RAD51-AS1 possibly forms a double-stranded structure with the 5′ UTR of RAD51 mRNA that may interfere with the binding of the ribosome or initiation factors and inhibit RAD51 translational initiation, thereby decreasing the DNA damage repair capacity of cells. Our previous study shows that RAD51-AS1 can inhibit the resistance of liver cancer cells to treatment and that this effect is different from the carcinogenic role of RAD51-AS1 described in the above study on ovarian cancer. We hypothesize that RAD51-AS1 participates in other physiological regulatory pathways and plays different regulatory roles in different cancer cell lines.

Studies have noted that the formation of a double-stranded structure between antisense lncRNA and complementary mRNA can trigger RNA interference by causing RNase H to recognize this double-stranded structure and cleave it, leading to mRNA degradation [42,43,44]. Moreover, some anti-sense RNA can block ribosome binding and inhibit initiation of translation [45,46]. Alternatively, antisense lncRNA, such as PCNA-AS1, can stabilize mRNA and promote protein expression [47]. Regarding why the two antisense lncRNAs have different regulatory mechanisms causing opposite results, it is hypothesized that this discrepancy is due to the length and site of the complementary sequence. Nonetheless, further studies are needed to elucidate the length of the double-stranded RNA required to attract RNase H.

RAD51 is an important protein that regulates repair of double-stranded DNA damage, and transcription factors such as p53 and E2F1 can regulate expression of this protein [48,49]. In the present study, we found that melatonin was able to suppress RAD51 mRNA expression, as recently reported [50]. However, we did not find that melatonin can regulate the abovementioned transcription factors to inhibit RAD51 expression; rather, protein repression was achieved through lncRNA RAD51-AS1. This mechanism is a novel regulatory pathway, and we theorize that it exists in other cancers. Accordingly, we suggest that melatonin regulates RAD51 expression at both the transcription and translation levels. Regardless, further studies are needed to determine how melatonin induces RAD51-AS1 expression. We hypothesize that melatonin may regulate RAD51-AS1 expression via transcriptional regulation or epigenetic mechanisms. Accordingly, we will investigate the relationship between melatonin and transcription factors regulating expression of RAD51-AS1 or the influence of melatonin on the epigenetic modification of the RAD51-AS promoter in future studies. As there are many molecules or ncRNAs in addition to RAD51that participate in the DNA damage repair process [51,52,53,54], another aim of our subsequent studies will be to determine whether melatonin participates in the regulation of these molecules.

Our rescue experiment (Figure 6) showed that knockdown of RAD51-AS1 expression leads to incomplete recovery of the anti-HCC phenotype mediated by melatonin. This suggests that other molecules (such as lncRNA-CPS1-IT1) are involved in melatonin’s regulation of cancer [55]. Further research is needed to confirm this.

Melatonin has anticancer effects on many types of cancer [56,57,58]. Nonetheless, relevant studies on liver cancer are scarce. In addition, only a few studies have reported a regulatory relationship between melatonin and lncRNA. In the present study, melatonin was confirmed to have inhibitory effects on HCC and to suppress the DNA damage repair capacity of HCC cells through lncRNA RAD51-AS1-mediated regulation of RAD51. In addition, melatonin was found to not be toxic and to hold promise as an adjuvant therapy in HCC treatment to increase the effectiveness of chemotherapy and radiotherapy.

## 4. Materials and Methods

### 4.1. Analysis of Melatonin, RAD51 and RAD51-AS1 Levels in Human Specimens

The human serum and fresh frozen tissues used in this study were obtained from 33 HCC patients who underwent surgical resection at Chang Gung Memorial Hospital (Tao-yuan, Taiwan) between 2010 and 2015. Serum melatonin levels were measured using an enzyme-linked immunosorbent assay (ELISA) kit (CUSABIO, Baltimore, MD, USA) according to the manufacturer’s instructions. RAD51 and RAD51-AS1 expression levels in the above tissue samples were analyzed by quantitative real-time RT-PCR using a TaqMan gene expression assay (Thermo Fisher Scientific, Waltham, MA, USA). To reduce differences between individuals, patients of the same sex and similar age and acquisition time were selected to rule out the influence of these factors. This study was approved by the Ethics Committee of Chang Gung Memorial Hospital (IRB approval no.: 201601767B0, approval date: 5 January 2017), and written informed consent was obtained from each patient.

### 4.2. Cell Lines, Antibodies, Drug, siRNA and Plasmid Construction

The HCC cell lines Huh7 and HepG2 were purchased from American Type Culture Collection (Manassas, VA, USA), which supplies authenticated cell lines. The cell lines were subjected to routine testing to confirm the absence of mycoplasma and cultured in DMEM medium containing 10% fetal bovine serum at 37 °C in a 5% CO_2_ atmosphere. Polyclonal antibodies against RAD51, cleaved caspase-3, cleaved caspase-9, MT1, MT2, ATM, ATR, RPA32 and β-actin were purchased from Cell Signaling Technology (Beverly, MA, USA) and Genetex (Irvine, CA, USA). Secondary antibodies were purchased from Santa Cruz Biotechnology (Santa Cruz, CA, USA). Commercialized si-RAD51-AS1 and negative-control siRNAs were purchased from Thermo Fisher Scientific. Melatonin and etoposide (VP16) powder (characterized by purity above 98%, as measured by TLC) were purchased from Sigma-Aldrich (St. Louis, MO, USA). A CMV-based expression plasmid containing lncRNA-RAD51-AS1, namely, pCDNA3.1-RAD51-AS1, was constructed by BIOTOOLS CO., LTD. (Taipei, Taiwan). The pCMV-FLAG-RAD51 plasmid, DR-GFP vector, and I-*Sce*I expression vector (for the HR assay) were kindly provided by Professor Chin-Chuan Chen.

### 4.3. Detection of lncRNA-RAD51-AS1 and RAD51 Levels Using Quantitative Real-Time RT-PCR

Total RNA from each tissue or cell line was isolated using an RNeasy mini kit (QIAGEN, Gaithersburg, MD, USA) according to the manufacturer’s instructions. Two micrograms of each RNA sample was reverse transcribed. These products were subjected to quantitative real-time RT-PCR to detect lncRNA-RAD51-AS1 and RAD51 expression using the TaqMan gene expression assay (Applied Biosystems, Foster City, CA, USA); GAPDH was used as an internal control.

### 4.4. Transfection and Western Blotting Analysis

Huh7 and HepG2 cells were seeded in 6-well plates at a density of 3 × 10^5^ cells/well and incubated overnight. The cells were transfected with 1 μg of plasmid (pCDNA3.1-RAD51-AS1 or pCDNA3.1 vector) or 50 nM siRNA (si-RAD51-AS1 or si-CTR) using Lipofectamine 2000 (Invitrogen, Carlsbad, CA, USA) according to the manufacturer’s instructions. Forty-eight hours later, the transfected cells were washed twice with PBS and then lysed in 200 μL of RIPA lysis buffer (BIOTOOLS CO., LTD., Taipei, Taiwan) containing protease inhibitors. Proteins (100 μg) from the supernatant were separated by SDS polyacrylamide gel electrophoresis, followed by western blotting analysis to detect levels of RAD51 and β-actin. The immunoreactive bands were visualized using an ECL system (NEN Life Science Products, Boston, MA, USA) and developed using X-ray films. The content of each band was quantified using ImageQuant 5.2 (GE Healthcare, Piscataway, NJ, USA).

### 4.5. Cell Proliferation Assay

Cell proliferation capacity was examined with an xCELLigence real-time cell analyzer (Roche Life Science, Indianapolis, IN, USA) and by a colony formation assay, as previously described [55].

### 4.6. Cell Migration and Invasion Assays

Cell migration activity was analyzed using a wound-healing assay and a transwell migration assay, as previously described [59].

### 4.7. Apoptosis Assay

The apoptosis status of Huh7 cells were determined using trypan blue exclusion assay and DeadEnd^TM^ Fluorometric TUNEL assay kit (Promega, Madison, WI, USA) according to the manufacturers’ protocol. In brief, Huh7 cells were treated with 1 mM melatonin, 200 µM etoposide (VP16), or both for 48 h. The cells were then subjected to a Terminal deoxynucleotidyl transferase dUTP nick end labeling (TUNEL) assay or stained with 0.04% (*w*/*v*) trypan blue solution (Invitrogen Life Technologies), which labels dead cells in blue, for 5 min at room temperature. The cells were then counted under microscopy (magnification, ×40); cells in five different fields of vision/dish were analyzed for each experiment.

### 4.8. Comet Assay

A comet assay was performed as described previously [41]. In brief, Huh7 cells were seeded in 24-well plates (4 × 10^4^ cells per well), incubated overnight, and treated with 200 µM etoposide for 1 h. The etoposide was then washed out with PBS, and the cells were treated with 1 mM melatonin or DMSO in etoposide-free medium. Four hours after treatment, the cells were harvested and subjected to the comet assay. Comet images were obtained using a fluorescence microscope (Nikon ECLIPSE Ni-U plus), and the tail moment was calculated using OpenComet software.

### 4.9. Immunofluorescence Staining of RAD51

Huh7 cells were treated with 200 µM etoposide for 1 h to induce DNA damage. The etoposide was then washed out, and the cells were allowed to recover in medium with or without 1 mM melatonin. At various time points, the cells were processed for immunofluorescence staining as described previously to detect the formation of RAD51 foci [60]. Slides were mounted in Vectashield containing DAPI (4′,6-diamidino-2-phenylindole; Vector Laboratories, Burlingame, CA, USA) and visualized using confocal microscopy (LSM 700; Carl Zeiss, Jena, Germany).

### 4.10. HR Assay

To determine the percentage of homologous recombination, a sample of 5 × 10^5^ cells was co-transfected with 1 µg of pDR-GFP (a gift from Maria Jasin, Addgene plasmid #26475) and I-*Sce* I (a gift from Maria Jasin, Addgene plasmid #26477) plasmids and treated with or without melatonin for 48 h. The cells were then trypsinized, washed once and resuspended in PBS. The percentage of GFP-positive cells was quantitated using a FACSCalibur device (Becton-Dickinson, San Jose, CA, USA).

### 4.11. Whole-Transcriptome Sequencing

Total RNA from Huh7 and HepG2 cells treated with and without 1 mM melatonin for 48 h was isolated using TRIzol reagent (Invitrogen) and checked for quality with the Bioanalyzer 2100 system (Agilent Technologies, Santa Clara, CA, USA). The qualified RNA was subjected to whole-transcriptome sequencing, as described previously [61].

### 4.12. Mice

Six week-old male nude mice (BALB/cAnN-Foxnlnu/CrlNarl) were purchased from the National Laboratory Animal Center (Taipei, Taiwan), housed under pathogen-free conditions and fed autoclaved standard chow and water. The mice were bred at the animal center of Chang Gung Memorial Hospital according to the Guidelines for the Care and Use of Laboratory Animals (NIH). All animal experiments were approved by the Institutional Animal Care and Use Committee (IACUC) of Chang Gung Memorial Hospital (IACUC approval no.: 2016121301, approval date: 24 February 2017).

### 4.13. Xenograft Assays and Drug Administration

A sample of 5 × 10^6^ Huh7 cells was resuspended in 100 μL of saline with 50% Matrigel (BD Biosciences) and subcutaneously implanted into the left and right flank regions of mice. All the tumors were staged for 1 week before drug treatment was initiated. At the beginning of the second week, mice with tumors were intraperitoneally (IP) injected with 100 µL of melatonin (at a dose of 40 mg/kg of body weight), etoposide (40 mg/kg) or an equal volume of dimethyl sulfoxide (DMSO), which served as a control, five days per week. The abovementioned drugs were administered 1 h before the room lights were switched off. Tumor volumes were measured three times per week using digital calipers.

### 4.14. Immunohistochemical Staining

The tumors of mice were fixed in formalin and embedded in paraffin. Consecutive sections (2 μm thick) were cut and subjected to immunohistochemical staining, as described previously [41].

### 4.15. Statistical Analysis

Original real-time PCR data and western blotting and migration assay analyses were considered to be continuous variables and analyzed using Student’s *t*-test. All statistical analyses were performed using SPSS 16.0 (IBM, New York, NY, USA) and Excel 2007 (Microsoft, Seattle, WA, USA). All statistical tests were two-sided, and *p*-values were considered significant at <0.05 (*), <0.01 (**), or <0.001 (***).

## 5. Conclusions

Melatonin inhibits the DNA repair capacity of HCC cells via lncRNA RAD51-AS1-mediated RAD51 suppression. Melatonin can be considered a promising adjuvant for chemotherapy and radiotherapy in HCC.

## Supplementary Materials

The following are available online at http://www.mdpi.com/2072-6694/10/9/320/s1, Figure S1: Differential expression of the melatonin receptor and RAD51 protein in hepatoma cell lines; Figure S2: Melatonin suppressed the colony formation ability of HCC cells; Figure S3: Melatonin enhanced etoposide (VP16)-induced apoptosis of Huh7 cells; Figure S4: Melatonin suppressed expression of DNA repair-related proteins; Figure S5: Whole-transcriptome sequencing analysis of different genes after melatonin treatment.
