# Supplementary material for: Melatonin Sensitizes Hepatocellular Carcinoma Cells to Chemotherapy Through Long Non-Coding RNA RAD51-AS1-Mediated Suppression of DNA Repair
Source: Cancers (Basel). 2018 Sep 10;10(9):320. doi: 10.3390/cancers10090320 (PMC6162454; doi:10.3390/cancers10090320)
Supplement: Supplementary file 1 [file cancers-10-00320-s001.docx]

Melatonin Sensitizes Hepatocellular Carcinoma Cells to Chemotherapy Through Long Non-coding RNA RAD51-AS1-Mediated Suppression of DNA Repair

Supplementary Materials

**
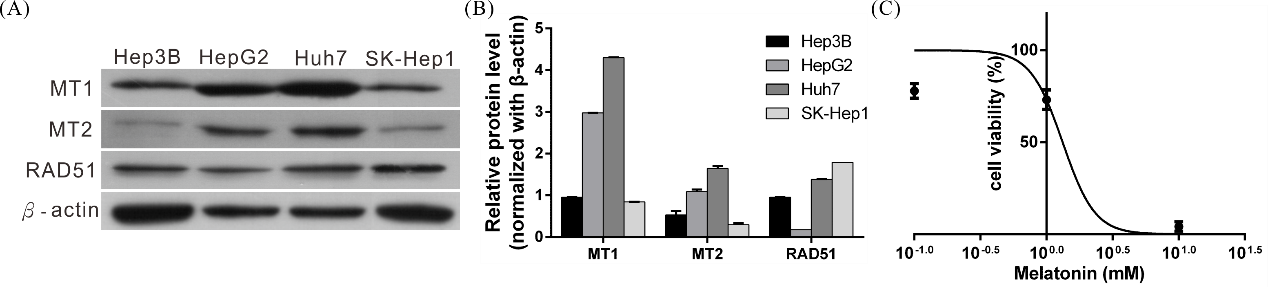
**

**Figure S1.** Differentially expression of melatonin receptor and RAD51 protein in hepatoma cell lines. (**A**) Western blot analysis showed the expression level of melatonin receptor (MT1 and MT2) and RAD51 in different hepatoma cell lines. β-actin was served as an internal control. The quantitative results are shown in (**B**). (**C**) The half-maximal inhibitory concentration (IC_50_) of melatonin was found to be 1.4 mM.


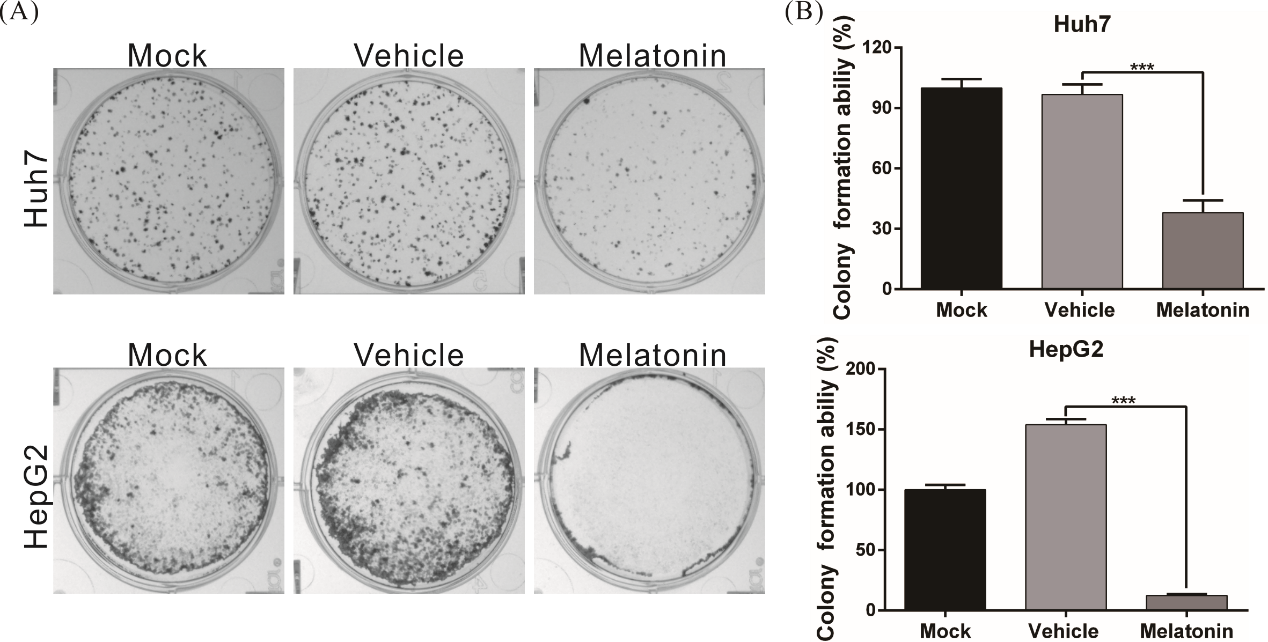


**Figure S2.** Melatonin suppressed colony formation ability of HCC cells. (**A**) Colony formation ability was analyzed in Huh7 and HepG2 cells treated with or without 1mM melatonin. The quantitative results are shown in (**B**). *p* < 0.001 (***).

**
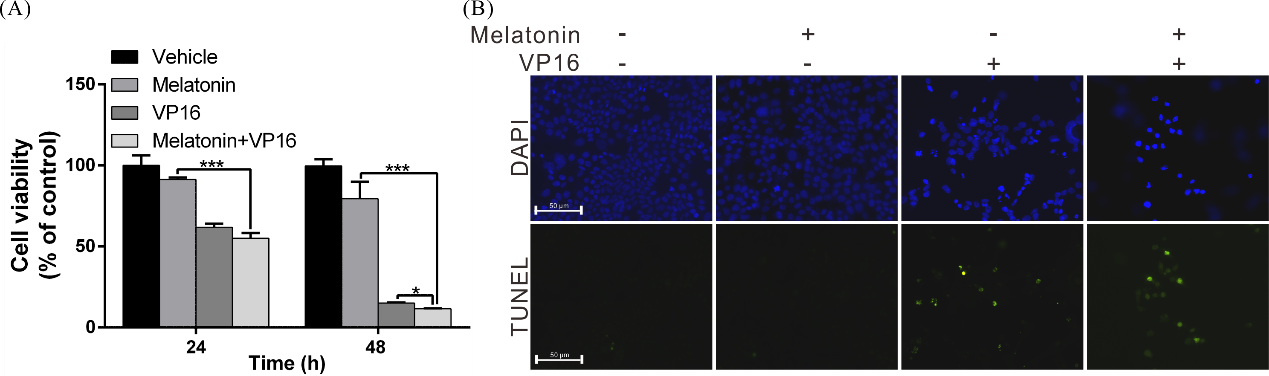
**

**Figure S3.** Melatonin enhanced etoposide (VP16)-induced apoptosis of Huh7 cells. Huh7 cells were treated with 1 mM melatonin, 200 µM etoposide (VP16), or both for 48 h, and then subjected to MTT assay (**A**) and TUNEL assay (**B**). Green punctate staining represented TUNEL-positive cells; apoptotic cells were identified with DAPI and TUNEL double stained cells. *p* < 0.05 (*), *p* < 0.001 (***).

**
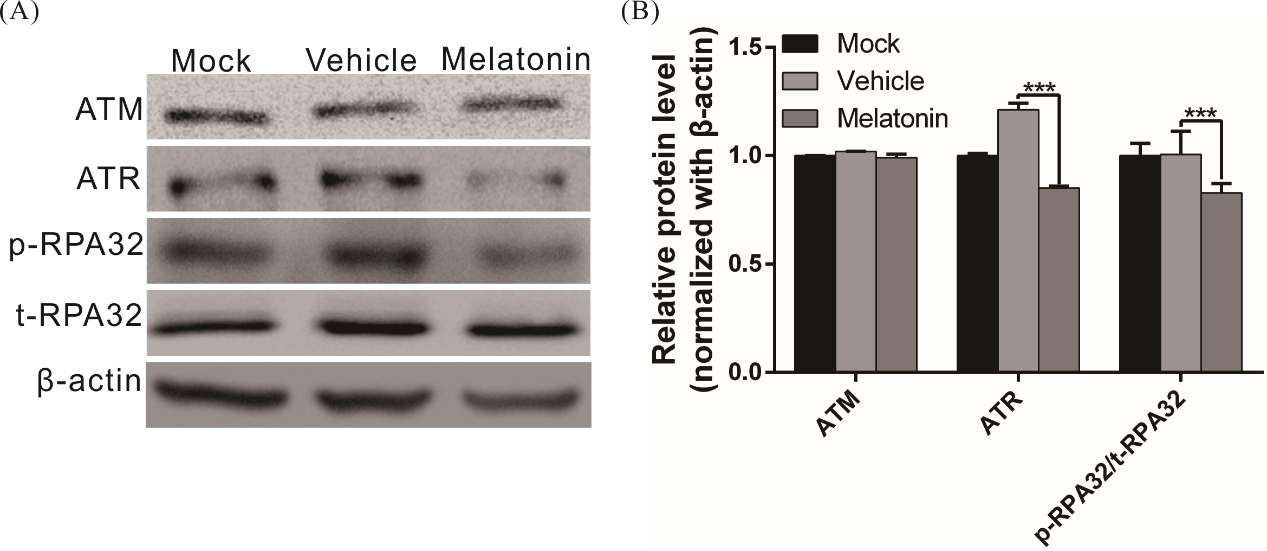
**

**Figure S4.** Melatonin suppressed the expression of DNA repair related proteins. (**A**) Western blot analysis of DNA repair related proteins after treated with/without 1 mM melatonin for 48 h. β-actin served as an internal control. The quantitative results are shown in (**B**). *p* < 0.001 (***). p-RPA32: phospho-RPA32; t-RPA32: total RPA32.


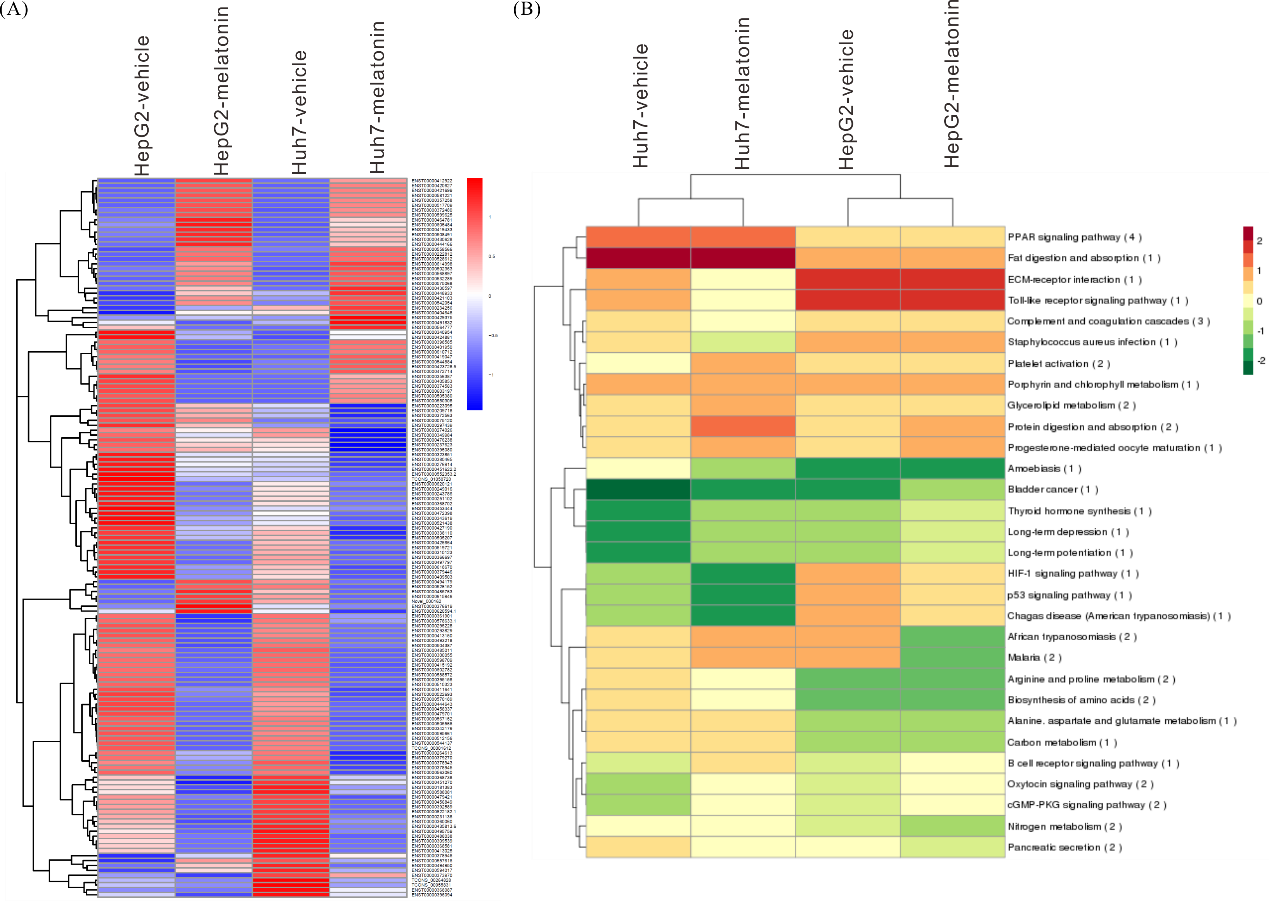


**Figure S5.** Whole-transcriptome sequencing analysis of different expression genes after melatonin treatment. (**A**) Heatmap comparing significant differentially expressed genes in Huh7 and HepG2 cell treated with or without 1 mM melatonin for 48 h. (**B**) Barcharts represent the enriched biological processes and biological pathways.


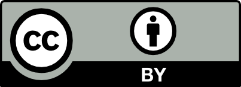
© 2018 by the authors. Licensee MDPI, Basel, Switzerland. This article is an open access article distributed under the terms and conditions of the Creative Commons Attribution (CC BY) license (http://creativecommons.org/licenses/by/4.0/).
